# A Treatise on Pharmacy, Designed as a Textbook for Students and a Guide for the Physician and Pharmacist

**Published:** 1884-04

**Authors:** 


**A Treatise on Pharmacy,** designed as a text-book for students and as a guide for the Physician and Pharmacist, containing the official and many unofficial formulas. By EDWARD PARISH, late Professor of Pharmacy in the Philadelphia College of Pharmacy. Fifth edition, enlarged and thoroughly revised by THOS. S. WIGAND, Ph. D., with 256 illustrations. Philadelphia: H. C. Lea's Son & Co. 1884.

It is only necessary to announce the appearance of a fifth edition of this standard work. In its special field it stands unequalled in any language. Every pharmacist of any education is familiar with it, and we would heartily commend it to every physician who is obliged to compound his own medicine. The book is presented in exceedingly handsome style by the publishers.

